# Peripheral Endothelial (Dys)Function, Arterial Stiffness and Carotid Intima-Media Thickness in Patients after Kawasaki Disease: A Systematic Review and Meta-Analyses

**DOI:** 10.1371/journal.pone.0130913

**Published:** 2015-07-10

**Authors:** Sanne M. Dietz, Carline E. A. Tacke, Barbara A. Hutten, Taco W. Kuijpers

**Affiliations:** 1 Department of pediatric hematology, immunology and infectious diseases, Emma Children’s Hospital, Academic Medical Center (AMC), Amsterdam, The Netherlands; 2 Department of Clinical Epidemiology, Biostatistics and Bioinformatics, AMC, Amsterdam, The Netherlands; Kaohsiung Chang Gung Memorial Hospital, TAIWAN

## Abstract

**Background:**

Kawasaki disease (KD) is a systemic pediatric vasculitis. Its main complication is the development of coronary arterial aneurysms (CAA), causing an increased risk for ischemia and myocardial infarction. It is unclear whether KD patients, apart from the presence of CAA, have an increased cardiovascular disease (CVD) risk due to the previous systemic vasculitis. The aim of this study was to systematically review and meta-analyse the literature regarding surrogate markers for CVD risk in KD patients.

**Methods:**

Medline and Embase were searched for articles comparing endothelial dysfunction (flow-mediated dilation, nitroglycerin-mediated dilation and peripheral arterial tonometry), vascular stiffness (stiffness index, pulse wave velocity) and carotid intima-media thickness (cIMT) between patients and controls. Two investigators assessed the articles for eligibility and evaluated quality.

**Results:**

Thirty studies were included. For all outcomes, moderate to high heterogeneity between studies was found. Most studies reported a decreased flow-mediated dilation in the whole KD- and CAA-positive group compared to controls, while data on CAA-negative patients were conflicting. The stiffness index was increased in the majority of studies evaluating the whole KD- and CAA-positive group, but not in most studies on CAA-negative patients. Mean cIMT was neither significantly increased in the whole KD-group nor in the CAA-positive group nor in most studies studying CAA-negative patients. Studies measuring maximum cIMT were conflicting.

**Conclusion:**

Literature suggests that surrogate markers for CVD risk in KD patients are increased in CAA-positive but not in CAA-negative patients. This may indicate that CAA-positive patients should be monitored for CVD in later life. The results of this review have to be interpreted with care due to substantial heterogeneity between studies and methodological limitations, as well as the lack of long-term follow-up studies.

## Introduction

Kawasaki disease (KD) is a pediatric vasculitis mainly affecting children under the age of 5[1]. Coronary artery aneurysms (CAA) develop in 25% of untreated and 5–15% of patients treated with intravenous immunoglobulins, making it the most common cause of pediatric acquired heart disease in the Western world.

It can be hypothesized that, due to the previous systemic vasculitis, patients with KD have an increased risk for cardiovascular disease (CVD) at a later age, apart from the presence or absence of CAA. This hypothesis is difficult to test since KD was first described less than 50 years ago and therefore most of the KD patients are too young to have experienced cardiovascular events.

In recent years, several non-invasive surrogate markers of CVD risk have become available.

Endothelial dysfunction can be measured by flow-mediated dilatation (FMD), nitroglycerin-mediated dilation (NMD) or peripheral arterial tonometry (PAT) [2,3]. Peripheral arterial stiffness can also be an indicator of increased CVD risk. It can be measured by pulse wave velocity (PWV) or by the beta stiffness index (SI) [4]. Furthermore, structural changes in the arterial wall can be found by measuring the carotid intima-media thickness (cIMT), well-established surrogate marker of atherosclerosis and subsequent predictor of cardiovascular events [5,6].

The aim of this study was to systematically review and meta-analyze the existing literature regarding CVD risk after KD, as measured by surrogate markers.

## Methods

### Search strategies

We conducted a systematic literature search of Medline (1966-September 2014) and Embase (1980-September 2014) for studies addressing KD and surrogate markers of cardiovascular risk (i.e. endothelial dysfunction, peripheral arterial stiffness and cIMT). We used two domains of MeSH terms and free text words combined by ‘AND’, and in each domain the terms were combined by ‘OR’. The first domain contained terms of KD (including all synonyms and abbreviations), and the second contained terms of surrogate markers of cardiovascular risk (including all synonyms, abbreviations and free word text such as ‘carotid intima-media thickness’, ‘vascular stiffness’, ‘endothelial dysfunction’, ‘flow-mediated dilatation’, ‘pulse wave velocity’, ‘peripheral arterial tonometry’). The complete protocol is registered in the Prospero database under CRD42014005706, the PRISMA checklist and Medline electronic search strategy are added as S1 PRISMA Checklist and S1 File.

### Study selection and quality assessment

We selected those original studies that reported on surrogate markers of cardiovascular risk (i.e. endothelial dysfunction [FMD, NMD, PAT], vascular stiffness [PWV, SI] and cIMT) in KD patients. Studies were excluded if healthy control groups were not available within the same studies, if lipid-lowering medication was used when measuring subjects, or if data contained preliminary results. Furthermore, because of the possible influence of the acute inflammation, studies measuring patients within 6 months after the acute phase were excluded. Language restrictions were not imposed. The selection process was divided into three successive stages: title-, abstract- and manuscript selection. Two investigators (SD and CT) independently determined eligibility of the retrieved studies, according to predefined criteria. Using an adjusted version of the Newcastle-Ottawa scale for observational studies (S1 Table: Quality assessment criteria), the same investigators assessed the methodological quality of the eligible studies. Selection of patients and controls, comparability, and outcome measurements were evaluated. Disagreements were solved by discussion, and if necessary, by the opinion of a third reviewer.

### Data extraction

Using a predetermined form, two investigators (SD and CT) independently extracted data of the eligible articles. Information was collected on study characteristics (study design, country and sample size). The CAA-classification used was retrieved and the number of CAA-positive patients was noted based on whether patients ever had CAA (worst-ever CAA-score). In addition, the following characteristics of participants were extracted: gender, age, blood pressure, BMI, and treatment during the acute KD phase. Outcome measurements were, if possible, collected for the control- and the whole KD group, as well for the CAA-negative and CAA-positive group. When data were missing, the corresponding authors were emailed to request the information.

### Statistical analysis

When studies described multiple CAA-positive groups based on severity, we calculated pooled estimates of the mean and standard deviation (SD) values for the overall CAA positive group. The same was done when cIMT was described separately for the left and the right carotid artery. When p-values were not provided, but mean, SD and numbers were reported, we calculated whether there was a significant difference between patients and controls using review manager software, version 5.2 (Cochrane Collaboration).

We used the same software to create forest plots for cIMT, FMD and SI in KD patient compared to controls and in CAA positive patients compared to controls, with study-level effect sizes calculated as absolute mean differences. We measured the proportion of between-study differences not attributable to chance with the I^2^ statistic. We considered values of 25–50%, 50–75% and ≥75% to indicate low, moderate and high heterogeneity, respectively. Only when heterogeneity was low or moderate (≤75%), pooled estimates of the summary mean difference were computed using the random-effects model according to the method of DerSimonian and Laird. [7]. A Z-test was performed to test the overall effect.

When studies with overlapping inclusions were present, the largest study was used for the forest plot and if applicable, the pooled estimate.

Heterogeneity was explored by a sensitivity analyses for all analyses. Furthermore, for the whole KD group, a meta-regression analysis using the random effects, methods of moments approach was performed, with study characteristics as covariates. We looked at ‘time since KD’, ‘percentage of IVIG-treated patients’ and ‘percentage of CAA-positive patients’. We did not perform meta-regression analyses on the CAA-positive groups because covariables were usually not described separately for CAA-positive groups in most studies. We used comprehensive meta-analyses software (version 2) to execute the meta-regression analyses.

We did not perform meta-analyses on the data of CAA-negative patients because not all studies used the same CAA-criteria. This implies that a child might have no enlargement according to one classification but does have an aneurysm according to the other. All CAA-positive patients have enlargement according to at least one classification system.

## Results

### Description of studies

**Fig 1:**
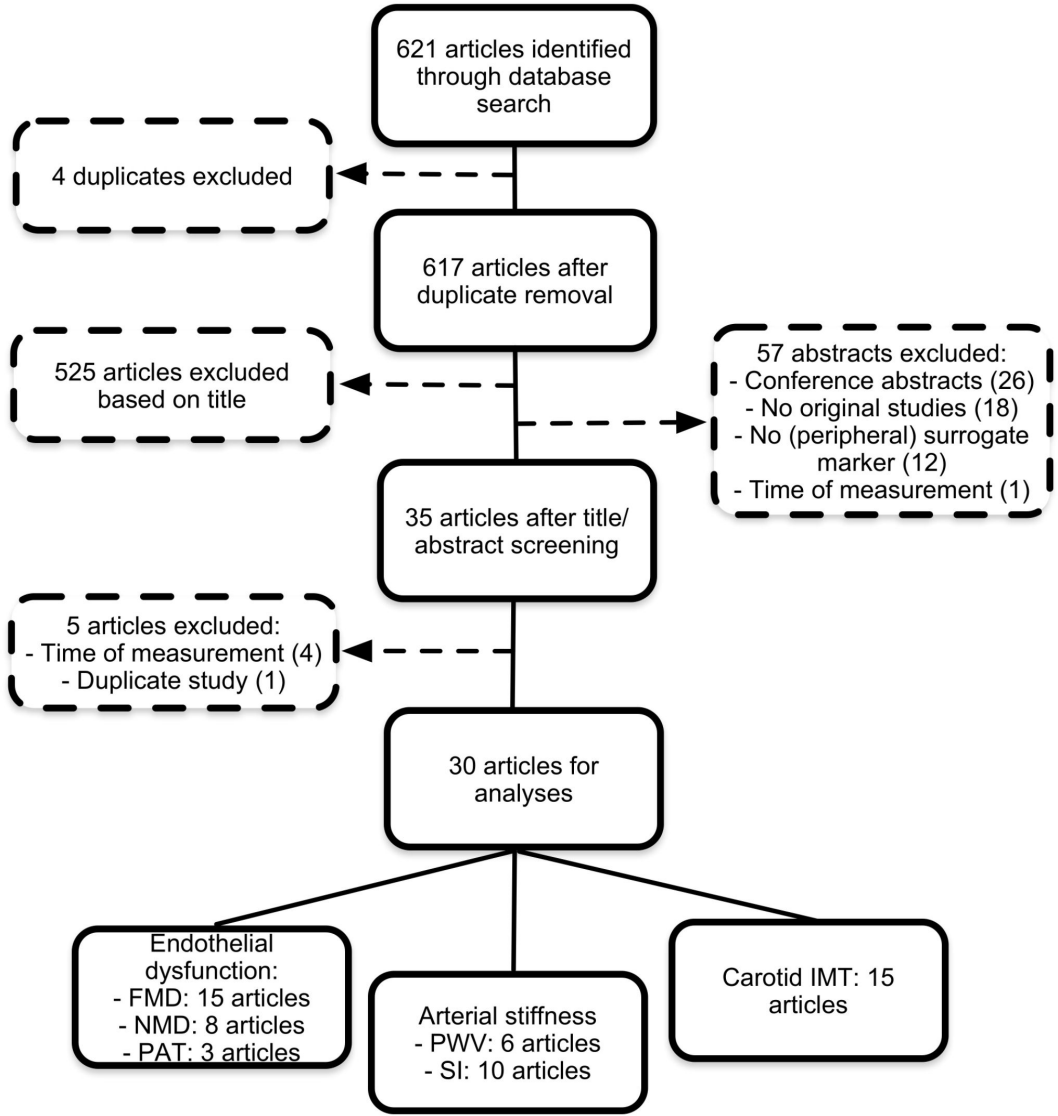
Flow diagram of selected studies.

Our search retrieved 621 articles. After scanning titles and/or abstracts, we excluded 586 studies. Of the remaining 35 articles, five were excluded based on the whole article. Hence, 30 studies remained for final inclusion (Fig 1). Table 1 shows the characteristics of these studies.

**Table 1:** Characteristics of studies.

First author, year	Country	Kawasaki patients					Controls			Reported outcome
		% IVIG	No; CAA+/CAA-	Age (yrs.)	% ♂	Time since KD (yrs.)	No.	Age (yrs.)	% ♂	
Dhillon, 1996 [8]	UK	15	20 (3/17)	13 (11–19)*	12 (60)	11.3 (5.3–17.1)*	20	15 (10–16)*	NA	FMD, NMD
Silva, 2001 [9]	Canada	33	24 (13/11)	14.3±1.8	18 (75)	11.3±1.8	11	14.1±1.5	6 (55)	FMD, NMD
Noto, 2001 [10]	Japan	58	20 (20/0)	16.6±4.1	12 (60)	9.8±4.0	20	16.3±4.7	12 (60)	cIMT, SI
Deng, 2002 [11]	China	87	39 (6/33)	7.1±2.7	28 (72)	3.4±2.1	17	7.0±3.1	13 (76)	FMD, NMD
Cheung, 2004 [12]	China	93	71 (43/28)	CAA+: 10.2±4.1	47 (66)	7.8 (1–16.6)†	35	10.3±3.6	25 (71)	SI
				CAA-: 9.7±3.2		6.9 (1.1–13.2)†				
Cheung, 2004 [13]	China	92	66 (37/29)	CAA+: 9.0±3.1	43 (65)	7.8±3.7	36	9.1±2.6	24 (67)	PWV
				CAA-: 8.9±3.2		6.2±2.4				
Cheung, 2004 [14]	China	93	71 (42/29)	9.5±3.7	48 (68)	7.7±3.9	41	10.5±3.7	28 (68)	PWV
Ikemoto, 2005 [15]	Japan	100‡	65 (34/31)	13 (9–22)*	38 (58)	12 (5–21)*	20	15 (9–23)*	11 (55)	cIMT, SI, FMD
Kadono, 2005 [16]	Japan	NA	24 (15/9)	8.3±4.1	13 (54)	5.8±4.6	41	10.7±4.4	29 (71)	cIMT, FMD
Borzutzky, 2007 [17]	Chili	100	11 (1/10)	10.6±2	7 (64)	8.1±3.6	11	10.4±1.8	7 (64)	FMD
McCrindle, 2007 [18]	Canada	64	52 (19/33§)	15.5±2.3	35 (67)	11.2±3.7	60	14.9±2.4	30 (50)	FMD, NMD
Cheung, 2007 [19]	China	90	50 (26/24)	CAA+:8.6±2.8	33 (66)	7.4±3.5	22	9.5±2.5	14 (64)	CIMT, PWV, SI
				CAA-:8.6±3.3		5.8±2.1				
Huang, 2008 [20]	Taiwan	100	11 (11/0)	12.9±2.5	8 (73)	10.77±3.01	11	12.97±2.42	8 (73)	FMD
Niboshi, 2008 [21]	Japan	6	35 (15/20)	27±4.2	16 (46)	24.1±4.5	36	25.5±3.9	19 (53)	FMD, PWV
Cheung, 2008 [22]	China	75	51 (32/19)	13.4±0.6	40 (78)	10.5±0.68	32	14.6±0.6	26 (81)	cIMT, SI
Liu, 2009 [23]	China	100	41 (21/20)	CAA+: 7.0 (3–11)†	25 (61)	5.0 (1.6–10.0)†	22	8.4 (3.2–14.0)†	13 (59)	FMD, SI
				CAA-: 7.3 (4.9–11.0)†		3.8 (1.5–8.0)†				
Gupta-Malhotra, 2009 [24]	USA	36	28 (9/19)	20.9±6	19 (68)	16±6	27	21.3±7.5	16 (59)	cIMT, SI
Lee, 2009 [25]	Korea	100	25 (25/0)	12.6±2.0	11 (44)	> 8	55	14.5±0.7¶	29 (53)	cIMT, PWV
Noto, 2009 [26]	Japan	52	35 (35/0)	20.5±9.3	28 (80)	18.6±8.4	35	19.6±7.2	28 (80)	cIMT, FMD, NMD, SI
Duan, 2011 [27]	China	97	31 (31/0)	6.2±3.4	22 (71)	2.53 (1–12.5)*	21	5.7±2.5	14 (67)	cIMT, FMD, NMD, SI
Noto, 2012 [28]	Japan	64	18 (18/0)	17.2±5.3	13 (72)	14.1±6.9	15	15.3±2.0	12 (80)	cIMT, SI
Pinto, 2013 [29]	Portugal	100	19 (0/19)	21±6	12 (63)	>5	16	21±6	9 (56)	PAT
Tobayama, 2013 [30]	Japan	21	14 (11/3)	31.5±5.5	8 (57)	28.6±5.6	41	32.6±5.3	21 (51)	PAT
Ishikawa, 2013 [31]	Japan	100	24 (9/15)	6.5±1.7	14 (58)	3.3 (2.0–4.4)||	22	7.9±2.8	13 (59)	cIMT, FMD, NMD
Selamet Tierney, 2013 [32]	USA	93	203 (47/156§)	16.73±4.21	122 (60)	11.6 (1.2–26)*	50	17.57±4.33	29 (58)	cIMT, PAT
Duan, 2014 [33]	China	100	13 (13/0)	5.8±2.1	13 (100)	3.1 ±1.7	14	5.5 ± 2.3	14 (100)	FMD, SI
Cho, 2014 [34]	Korea	91	68 (19/49)	CAA+: 8.00±1.89	40 (59)	5.75±2.65	30	7.65 ± 0.78	16 (53)	PWV
				CAA-: 7.22±1.49		4.36±2.21				
Laurito, 2014 [35]	Italy	0	14 (7/7)	10.0±3.7	9 (64)	6.3±4.8	14	10.2±2.4	7 (50)	cIMT, FMD
Oguri, 2014 [36]	Japan	81	75 (11/64)	8.2±2.8	49 (65)	5.68±2.48	50	8.3±3.5	25 (46)¶	cIMT, SI
Singh-Meena, 2014 [37]	India	100	27 (10/17)	8.22±2.6	20 (74)	2.45 ± 1.20	23	8.46 ± 2.9	12 (52)	cIMT

Values represent mean ± SD unless otherwise indicated.

* Median (range).

† mean (range).

‡ 400mg for 5 days

§ McCrindle et al: Ectasia in 8 patients. Selamet Tierney et al: Ectasia in 20 patients.

|| Median (IQR)

¶ p<0.05 for age or sex when KD group is compared to control group

IVIG, Intravenous immunoglobulin; CAA, Coronary arterial aneurysm; FMD, Flow-mediated dilation; NA, not available; NMD, Nitroglycerin-mediated dilation; cIMT, Carotid intima-media thickness; SI, stiffness index; PWV, Pulse wave velocity

Four studies were cohort studies with a maximal follow-up of 6 months [11,20,33,37] and the remainder had a cross-sectional study design. The total number of subjects per study varied from 22 to 253. The mean age of KD patients ranged from 6.5 to 31.5 years. Although the criteria used were not clearly stated in all manuscripts, most studies defined CAA according to Japanese criteria or by z-scores [38,39]. Two articles used deviating classifications [11,23]. Most studies reported the worst-ever CAA-score, two studies reported the CAA-status 30 days after beginning of the disease [15,36] and in three studies it was not clear which status was reported on [16,17,30]. Some research groups invited the same patients for different studies, which created overlap of inclusions between studies [10,12–14,19,22,26–28,33]. Table 2 shows a summary of findings.

**Table 2:** Summary of findings.

	Site, unit		Mean controls	Mean CAA- and CAA+ pt.	P *	Mean CAA-	*P* †	Mean CAA+	*P* ‡
FMD and NMD									
**Dhillon, 1996 [8]**	BA, side unclear	% FMD	9.40±3.5	3.1±3.5	<0.001	-	-	-	-
		% NMD	21.7±5.4	23.0±9.5	0.58	-	-	-	-
**Silva, 2001 [9]**	BA, side unclear	% FMD	6.20±2.8	4.6±3.3	0.17	-	-	-	-
		% NMD	15.1±3.6	14.4±6.9	0.73	-	-	-	-
**Deng, 2002 [11]**	Right BA	% FMD	14.1±6.8	6.2±3.9	<0.0001	6.3±4.3	-	5.7±1.4	-
		% NMD	33.2±13.7	30.6±9.2	0.49	30.2±9.7	-	33.3±5.0	-
**Ikemoto, 2005 [15]**	Right BA	% FMD	18.8±2.8	-	-	19.4±3.9	NS	*12*.*5±7*.*6*	§
**Kadono, 2005 [16]**	Right BA	% FMD	11.7±14.7	3±11.0	<0.05	8.3±9.1	-	-0.5±9.2	-
**Borzutzky, 2008 [17]**	BA, n-d arm	% FMD	8.00±2.9	11.1±5.7	0.12	-	-	-	-
**McCrindle, 2007 [18]**	BA, side unclear	% FMD	9.4±9.0	8.9±11.6	0.6	-	-	-	-
		% NMD	-	-	0.93	-	-	-	-
**Huang, 2008 [20]**	Right BA	% FMD	13.11±1.00	-	-	-	-	6.12±1.61	<0.001
**Niboshi, 2008 [21]**	Right BA	% FMD	14.4±3.2	10.4±2.6	<0.01	11.5±2.8	< 0.05	*9*.*1±2*.*1*	< 0.05
**Liu, 2009 [23]**	Left BA	% FMD	12.1±2.3	-	-	9.5±2.8	<0.01	4.5±1.5	<0.01
**Noto, 2009 [26]**	BA, side unclear	% FMD	13.30±4.8	-	-	-	-	9.1±2.7	<0.001
		% NMD	20.6±7.0	-	-	-	-	20.5±6.2	0.96
**Duan, 2011 [27]**	Left BA	% FMD	12±8	-	-	-	-	4±8	0.001
		% NMD	29±12	-	-	-	-	23±10	0.075
**Ishikawa, 2013 [31]**	Left BA	% FMD	11.10 (10.1–13.9)||	-	-	9.1 (6.6–10.7)||	<0.05	4.4 (2.6–5.7)||	<0.01
		% NMD	25.1±4.5	-	-	24.0±8.2	NS	21.7±5.0	NS
**Duan, 2014 [33]**	Left BA	% FMD	11.1 (8.8–16.4)||	-	-	-	-	4.2 (-1.71–8.16)||	0.001
		% NMD	24.13 (18.62–30.86)||	-	-	-	-	24.4 (11.2–30.65)||	0.545
**Laurito, 2014 [35]**	Right BA	% FMD	9.54±1.8	9.38 ± 1.4	0.79	9.5±1.2	-	9.3±1.6	-
**PAT**									
**Pinto, 2013 [29]**	Left and right index fingers		2.31±0.53	-	-	1.67±0.49	0.001	-	-
**Tobayama, 2013 [30]**	1 finger of each hand		1.89±0.51	2.03 ± 0.44	0.19	-	-	-	-
**Selamet Tierney, 2014 [32]**	Left and right index fingers		1.70±0.53	1.78 ± 0.46	0.35	1.79±0.44	0.55||	*1*.*71±0*.*51*	0.55||
**SI**									
**Noto, 2001 [10]**	Right CA		2.94±0.91	-	-	-	-	4.11±0.86	< 0.001
**Cheung, 2004 [12]**	Right CA		4.24±0.86	-	-	4.27±0.83	-	5.07±1.11	0.001
**Ikemoto, 2005 [15]**	CA, side unclear		2.47±0.79	-	-	2.54±0.9	NS	*2*.*59±1*.*06*	NS
**Cheung, 2007 [19]**	Right CA		3.77±0.92	-	-	4.22±0.64	-	4.72±1.20	0.003
**Cheung, 2008 [22]**	Right CA		3.39±0.76	-	-	3.79±1.00	-	4.38±1.10	< 0.001
**Liu, 2009 [23]**	Right CA		3.59±0.46	-	-	3.81±0.50	0.142	4.10±0.44	<0.01
**Gupta-Malhotra, 2009 [24]**	Left and right CA		1.93±0.48	1.9±0.1	0.912#	2.0±0.7	0.912#	1.8±0.3	0.912#
**Duan, 2011 [27]**	Right CA		2.8±0.6	-	-	-	-	3.6±0.8	0.001
**Oguri, 2014 [36]**	Right CA		2.89±0.59	3.03±0.61	0.2	-	-	-	-
**Duan, 2014 [33]**	Right CA		2.93 (2.67–3.15)	-	-	-	-	3.12 (3.02–3.37)	0.032
**PWV**									
**Cheung, 2004 [13]**	Right brachial-radial, m/s		5.89±1.35	-	-	6.71±1.82	0.042	7.17±1.79	0.001
**Cheung, 2004 [14]**	Right brachial-radial, m/s		6.2±1.5	7.2 ± 2.8	0.034	-	-	-	-
**Cheung, 2007 [19]**	Right brachial-radial, m/s		5.77±1.25	-	-	6.73±1.88	-	7.22±1.67	0.009
**Niboshi, 2008 [21]**	L+R brachial-ankle, cm/s		*1103±139*	*1146 ± 147*	¶	-	-	-	-
**Lee, 2009 [25]**	Left brachial-ankle, cm/s		984.0±96.5	-	-	-	-	1020.6±146.5	<0.05
	Right brachial-ankle, cm/s		976.3±93.5	-	-	-	-	979.7±154.8	NS
**Cho, 2014 [34]**	L+R brachial-radial, cm/s**		966.71±88.7	-	-	1076.86±164.10	< 0.05	1181.50±7.78	<0.05
**cIMT**									
**Noto, 2001 [10]**	Max RCCA, mm		0.48±0.08	-	-	-	-	0.54±0.09	<0.05
**Ikemoto, 2005 [15]**	Max RLCCA, mm		0.5±0.04	-	-	0.47±0.06	NS	*0*.*50±0*.*05*	NS
**Kadono, 2005 [16]**	Max RCCA, mm		0.46±0.06	0.45±0.07	0.77	-	-	-	-
**Cheung, 2007 [19]**	Mean RCCA, mm		0.36±0.04	-	-	0.39±0.04	0.008	0.41±0.04	<0.001
**Cheung, 2008 [22]**	Mean RCCA, mm		0.42±0.016	-	-	0.42±0.019	NS	0.44±0.023	0.006
**Gupta-Malhotra, 2009 [24]**	Mean RLCCA, mm		0.48±0.06	0.49±0.07	0.905#	0.5±0.01	0.905#	0.47±0.01	0.905#
**Lee, 2009 [25]**	Max RCCA, mm		0.50±0.01	-	-	-	-	0.41±0.19	NS
**Noto, 2009 [26]**	Max RCCA, mm		0.46±0.05	-	-	-	-	0.57±0.15	<0.001
**Duan, 2011 [27]**	Mean RCCA, mm		0.44±0.04	-	-	-	-	0.49±0.07	0.025
**Noto, 2012 [28]**	Max, RCCA, mm		0.42±0.04	-	-	-	-	0.54±0.08	0.005
**Ishikawa, 2013 [31]**	Mean RCCA, mm		0.43±0.04	-	-	0.43±0.02	NS	0.45±0.03	NS
**Selamet Tierney, 2013 [32]**	Mean RCCA, mm		0.432±0.029	0.428±0.024	0.35	0.429±0.02	0.41#	*0*.*426±0*.*024* ††	0.41#
	Mean LCCA, mm		0.434±0.028	0.438±0.034	0.42	0.439±0.04	0.05#	*0*.*439±0*.*035* ††	0.05#
	Mean LRCCA calculated		*0*.*433±0*.*028*	*0*.*433±0*.*030*		*0*.*434±0*.*032*		*0*.*433±0*.*031*	
**Singh-Meena, 2014 [37]**	Max LCCA, mm		0.417±0.065	0.500±0.071	<0.001	-	-	-	-
**Laurito, 2014 [35]**	Mean RLCCA, mm		0.5±0.1	0.5±0.1	0.93	0.4±0.1	-	0.5±0.1	-
**Oguri, 2014 [36]**	Mean RCCA, mm		0.39±0.04	0.40±0.03	0.15	-	-	-	-

* p whole KD group vs controls

† p CAA-negative patients vs controls

‡ p CAA-positive patients vs controls

§ p< 0.0001 moderate and severe aneurysms vs controls

|| Median (IQR)

# based on ANOVA

¶ p<0.05 for male patients, p not significant for female patients

** in original article stated as m/s

†† z-score > 3.

NS, non-significant (as reported in original articles); BA, Brachial artery; cIMT; carotid intima-media thickness; FMD, flow-mediated dilatation; LCCA, Left common carotid artery; n-d; non-dominant; NMD, nitroglycerin-mediated dilation; PAT, peripheral arterial tonometry; PWV, pulse wave velocity; RCCA, Right common carotid artery; SI, stiffness index. *Italic numbers are calculated using the data in the original articles*

### Quality of studies

**Fig 2:**
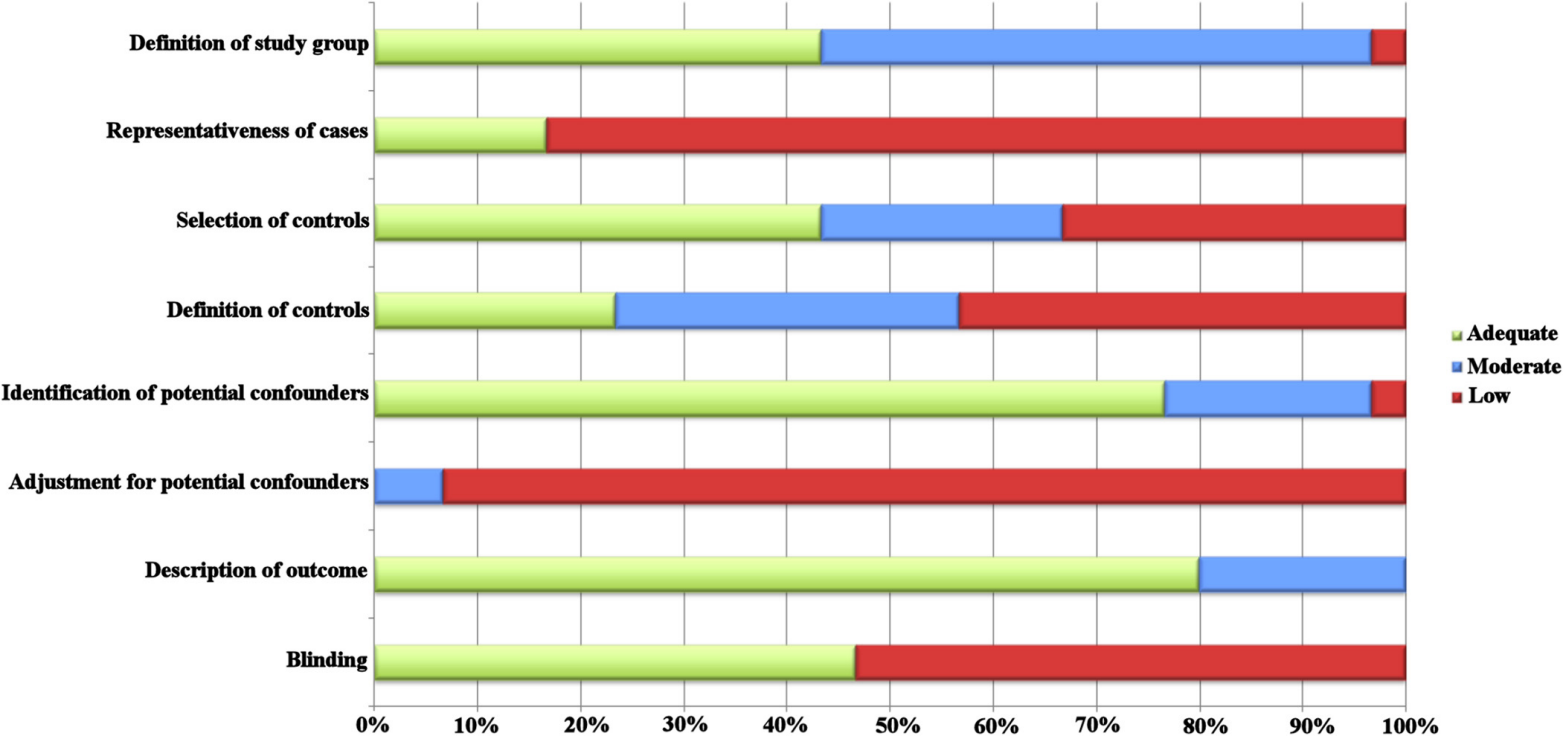
Quality of studies.

The results of the quality assessments are shown in Fig 2. All studies were found to have methodological limitations; the overall scores ranged from 4 to 11 (maximum of 16, S2 Table: Quality assessment per study). Most limitation arose from representativeness of cases, definition of controls and lack of adjustment for potential confounding factors.

### Endothelial dysfunction

#### Flow-mediated dilation

A total of 15 studies reported on FMD (Table 2). Eleven studies showed a significantly decreased FMD in patients after KD as compared to controls, with a mean or median difference ranging from -9.7% to -2.7%. Four studies showed no statistical significant difference.

**Fig 3:**
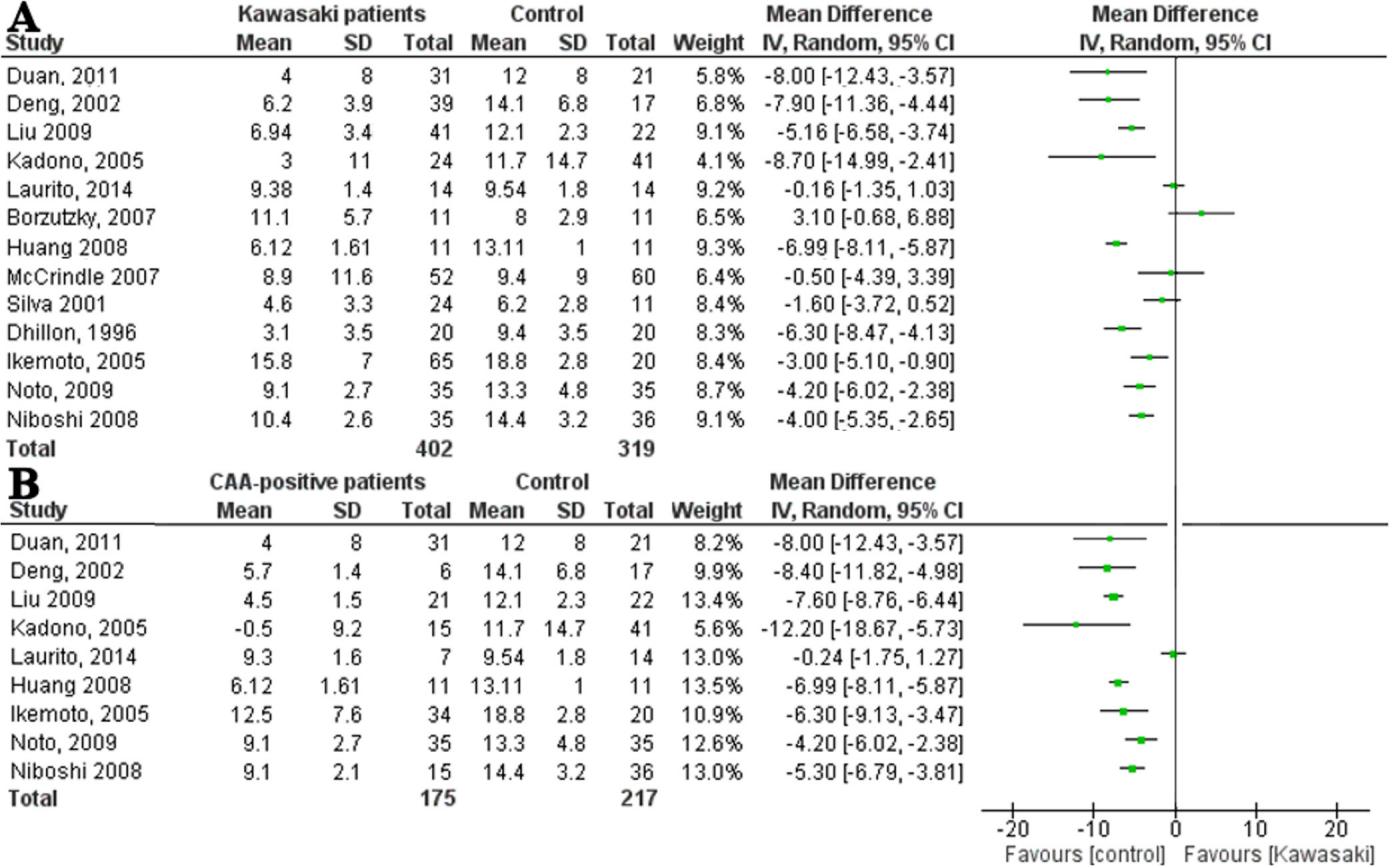
Forest plots of difference in flow-mediated dilation between patients and controls. **(A)** Forest plot: flow-mediated dilation of Kawasaki patients and controls, in order of ‘time since KD’. (**B)** Forest plot: flow-mediated dilation of CAA-positive patients and controls, in order of ‘time since KD’.

In meta-analyses, after excluding two studies reporting median instead of mean FMD, an extensive heterogeneity between the remaining 13 studies was found (I^2^ = 89%). Therefore, we did not pool the results (Fig 3A). Subsequently, heterogeneity was explored by sensitivity analyses and by means of meta-regression analysis. None of the three predefined covariables were shown to be of significant influence on heterogeneity.

#### Flow-mediated dilation and CAA status

Seven studies compared CAA-negative patients to controls; four studies found a significantly decreased FMD, whereas three studies did not find any difference (Table 2).

CAA-positive patients were found to have a significantly decreased FMD compared to controls in 10 out of 11 studies. Two studies reported median FMD. When analyzing the nine studies describing mean FMD, a high heterogeneity was found (I^2^ = 89%, Fig 3B). If the small study by Laurito *et al*. [35], which included only seven patients with mild transient CAA, was excluded from the analyses, moderate heterogeneity was calculated (I^2^ = 59%). Combining the remaining eight studies, a statistically significant decreased FMD was observed in patients after KD (mean difference -6.06%, 95%CI: -7.76% to -5.47%).

Niboshi *et al*. found that, compared to patients without CAA, FMD was significantly decreased in patients with persisting CAA but not in patients with transient CAA [21]. Ikemoto *et al* found a significant negative correlation between the severity of the coronary artery lesion and FMD [15].

#### Nitro-glycerine-mediated dilation

In addition to FMD, seven studies measured NMD (Table 2). None of the studies found a significant difference between patients after KD and controls, neither when looking at the groups as a whole nor when looking at CAA-negative or CAA-positive patients.

#### Peripheral arterial tonometry

All three studies reporting PAT used the Endo-PAT device to measure the reactive hyperemia index (RHI) or Endo-PAT Index. Selamet Tierney *et al*., studying 203 patients, found no difference in Endo-PAT index between patients and controls [32]. A similar result was reported by Tobayama *et al*.[30]. Pinto *et al*. found a significantly lower RHI in their CAA-negative patients (Table 2) [29].

### Vascular stiffness

#### Stiffness index

A total of 10 studies studied the SI of the carotid artery (Table 2). Seven studies found a significant increased SI in patients compared to controls, while three studies found no difference.

**Fig 4:**
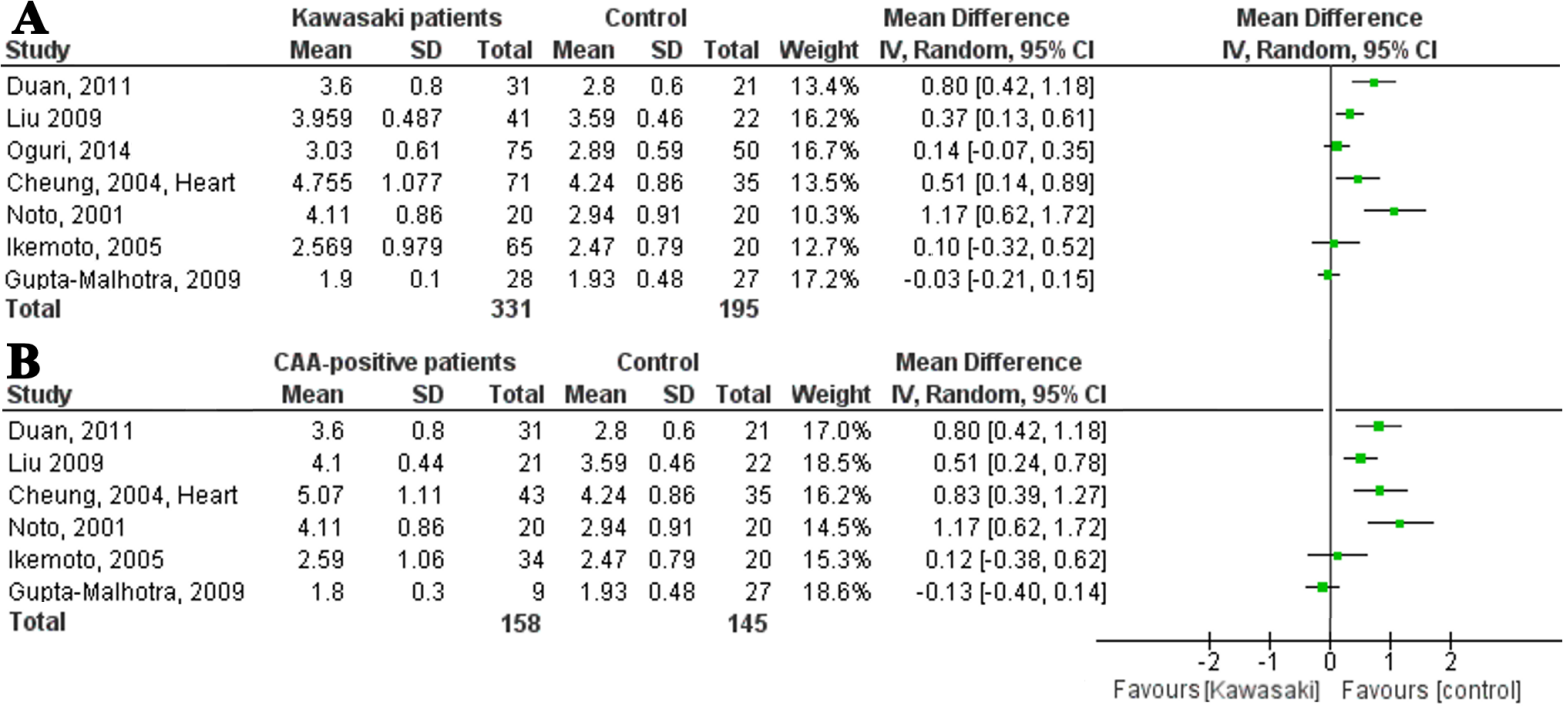
Forest plots of difference in stiffness index between patients and controls. **(A)** Forest plot: stiffness index of Kawasaki patients and controls, in order of ‘time since KD’. **(B)** Forest plot: stiffness index of CAA-positive patients and controls, in order of ‘time since KD’.

After excluding two studies because of overlapping inclusions in the meta-analysis [19,22] and one study because they reported the median [33], there was high heterogeneity between the remaining studies (I^2^ = 81%, Fig 4A).

A meta-regression analysis showed an independent positive association between the percentage of CAA-positive patients and the mean difference in SI (p<0.0001), which may partly explain the high heterogeneity.

#### Stiffness index and CAA status

Five out of six studies reporting on CAA-negative patients did not find a significant difference in SI of patients compared to controls.

Nine studies studied CAA-positive patients. Seven studies found a significant difference in SI between CAA-positive patients and controls, while two studies did not. Two studies were not included in the meta-analysis because of overlapping inclusions [19,22] and one because it measured the median instead of mean[33]. High heterogeneity was found between the remaining six studies (I^2^ = 88%; Fig 4B). After excluding the small study of Gupta-Malhotra *et al*.[24] with only nine patients with early transient CAA, heterogeneity decreased to 60%. When pooling the data of the remaining studies, a history of CAA was associated with a significantly increased SI of 0.67 (95% CI 0.38–0.96).

#### Pulse wave velocity

PWV in patients after KD was reported in six studies. Cheung *et al*. performed three of these, all showing an increased brachial-radial PWV in CAA-positive and CAA-negative patients compared to controls [13,14,19]. These results were similar to the results of Cho *et al*.[34].

Two studies measured brachial-ankle PWV (baPWV). Lee *et al*. found an increased PWV in their CAA-positive patients compared to controls [25]. Of note, their patient group was significantly younger than the control group, although one would expect the difference to be greater as PWV increases with age. Niboshi *et al*. found a significantly faster PWV in adult male but not in female KD patients as compared to controls [21].

### Carotid intima-media thickness

A total of 15 studies reported on cIMT in patients after KD (Table 2). The studies reported the cIMT of the right common carotid artery (CCA), the left CCA or the mean of both. Mean cIMT was reported in eight, while maximum cIMT was reported in seven studies.

Seven studies reported a significantly increased cIMT, seven studies showed no significant difference and one study showed a decreased cIMT as compared to controls.

**Fig 5:**
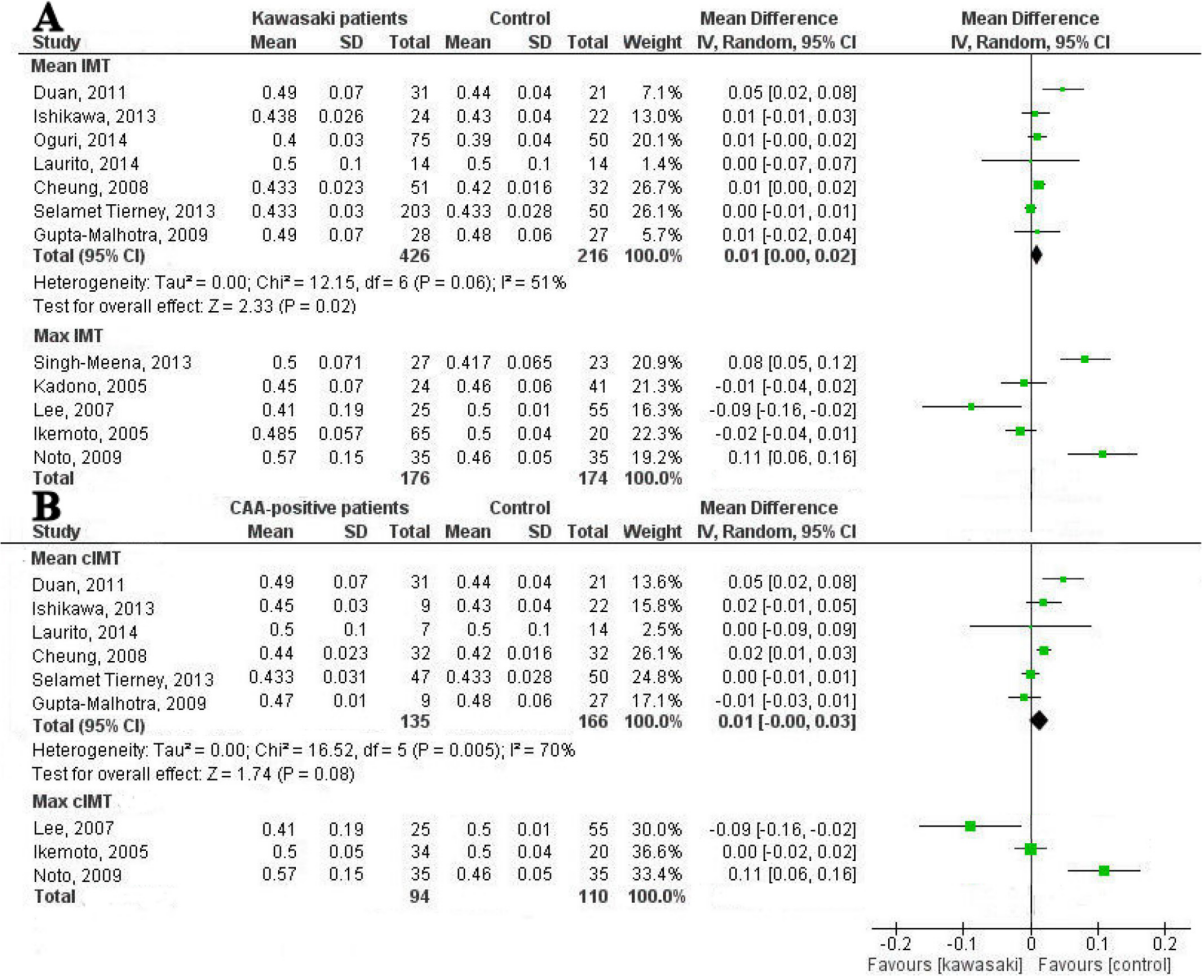
Forest plots of difference in carotid intima-media thickness between patients and controls. **(A)** Forest plot: Carotid intima-media thickness of Kawasaki patients and controls, in order of ‘time since KD’. (**B)** Forest plot: Carotid intima-media thickness of CAA-positive patients and controls, in order of ‘time since KD’.

In the meta-analysis, studies measuring mean cIMT and maximum cIMT were analyzed separately (Fig 5A and 5B). When analyzing the studies measuring mean cIMT, one study was excluded because of overlapping inclusions [19]. We found moderate heterogeneity in the remaining seven studies (I^2^ = 51%). When pooling the data of these studies, a mean difference of 0.01 mm (95% CI 0.00 to 0.02 mm) was found between patients and controls (Fig 5A). It was remarkable that in the two studies showing a thinner cIMT in KD patients, the control group was or seemed to be significantly older [16,25].

In meta-regression analyses none of the three predefined covariables was of significant influence on the heterogeneity.

Two studies measuring maximum cIMT were not included in the forest plot because of overlapping inclusions (Fig 5A) [10,28]. We did not combine the data of the five remaining studies because of the limited number of studies and the high heterogeneity and conflicting results (I^2^ = 90%).

#### Carotid intima-media thickness and CAA-status

Seven studies described CAA-negative patients and only one found an increased cIMT in patients compared to controls (Table 2).

Of the twelve studies measuring cIMT in CAA-positive patients, six reported a significantly increased cIMT and one showed a decreased cIMT (Table 2). One of the seven studies measuring mean cIMT was not included in the forest plot because of overlapping inclusions. The remaining six studies showed a moderate heterogeneity (I^2^ = 70%; Fig 5B). When pooling the data of these studies, a mean difference of 0.01 mm (95% CI 0.00 to 0.03 mm) was found between patients and controls (Fig 5B).

Maximum cIMT of CAA-positive patients was measured in five studies of which two were not included in the meta-analysis because of overlapping inclusions. As is shown in Fig 5B, the remaining studies had conflicting results.

Two studies divided their CAA-positive patients in subgroups according to diameter or z-score [15,32]. Ikemoto *et al*. did not find any difference between patients with mild, moderate and giant aneurysms, although these groups consisted of few patients only (16, 8 and 10 patients respectively). Selamet Tierney *et al*. found a significantly thicker left cIMT in patients with a history of giant aneurysms, whereas patients with a history of ectasia, small or medium CAA did not show this phenomenon.

## Discussion

Our systematic review summarizes 30 studies on surrogate markers for CVD risk in patients after KD compared to unaffected controls. FMD and SI were increased in most studies, being more pronounced in CAA-positive patients. Mean CIMT in the whole KD-group and the CAA-positive group did not seem to be increased while data on maximum cIMT were inconclusive. The results of this review have to be interpreted with care due to methodological limitations and substantial heterogeneity between studies.

### Quality of studies

Most studies had important methodological limitations. First, CVD risk is dependent on many factors. It has been shown that cIMT and FMD are dependent on life-style factors such as social-economic status and physical activity [40,41]. Therefore, a suitable control group is vital, which many studies failed to include or describe. Secondly, factors such as age, gender, blood pressure and BMI are known to influence surrogate markers [42]. These variables should be identified and adjusted for in the final outcome measurement. None of the studies adjusted for these factors. Moreover, most surrogate markers are highly dependent on the ultrasonographist(s) and/or interpreter(s) of the images. Hence, blinding is required but this was not described in 16 out of the 30 studies. Finally, many studies included a very limited number of patients. Only three studies included ≥50 participants for both groups.

### Heterogeneity of studies

Substantial heterogeneity existed between studies. In addition to the methodological limitations, the study populations varied in ‘time since KD’, ‘number of CAA-positive patients’, ‘percentage of IVIG-treated patients’, ‘gender distribution’, ‘age’, ‘ethnicity’ and ‘CAA-criteria’. When exploring heterogeneity by analyzing the first three variables, we could only find a significant covariate for the meta-analyses on SI. It was however, difficult to define some of these variables: the percentage of CAA-positive patients does not necessarily correlate to the severity of the aneurysms; some studies only included patients with transient dilations, while others included patients with severe or persistent aneurysms.

### Endothelial (dys)function

FMD in correlation with CVD risk has been researched extensively. Ras *et al*. found a CVD risk ratio of 0.9 per 1% higher FMD in a systematic review in adults [43]. In children, a significantly lower FMD has been found in sub-populations with an increased cardiovascular risk such as familial hypercholesterolemia [44].

FMD is an endothelium-dependent marker, which is mediated by the release of nitric oxide (NO) [45]. In contrast to FMD, NMD is an endothelium-independent marker, thought to reflect smooth muscle (dys)function. It has shown to be increased in diabetes mellitus and hypertension and is suggested to be a marker of the grade of cardiovascular risk[46]. NMD was not increased in any of the studies in this review, suggesting that the patients are at-risk but the endothelial dysfunction is at an early stage when smooth muscle function is not (yet) affected.

PAT is thought to correlate with coronary endothelial dysfunction. Studies in adults and children have shown a correlation between a lower PAT and coronary atherosclerosis and cardiovascular events or risk factors [47,48]. An earlier, large cohort study did not show correlation between FMD and PAT, indicating that they might reflect distinct aspects of endothelial function and possibly explaining the difference in PAT and FMD in our review [49].

### Arterial stiffness

Aortic PWV is a known predictor of cardiovascular events [50]. In contrast, studies included in this review measured brachial-radial PWV or baPWV. Earlier studies found a significant correlation between baPWV and cardiovascular events or risk factors, but large prognostic studies are missing [51,52]. Brachioradial PWV is less common in use and to our knowledge, no large studies looking at the association between brachioradial PWV and cardiovascular events have been performed.

SI has shown to be increased in children with obesity and in adults after myocardial infarction, although no large studies have investigated the exact correlation between CVD event and SI [53,54].

### Carotid IMT

CIMT is a validated measure of cardiovascular risk. Lorenz *et al*. found a hazard ratio (HR) of 1.15 for myocardial infarction (MI) and 1.18 for stroke with every 0.1 mm increase in cIMT in their systematic review [6]. In addition, they showed that people <50 years of age are at higher relative risk with increasing cIMT compared to people >50 years [55]. Also, hazards increase significantly faster for cIMT values of 0.6–1 mm compared to higher IMT values [56].

Although cIMT is validated, it is important to realize that a distance of 0.5 mm is measured using a device with an axial resolution of around 0.04–0.05 mm, implicating a large standard deviation by default, hence not suitable for research in small groups. In our review, 13 out of the 15 studies measuring cIMT included less than 50 participants per group.

### Cardiovascular disease risk

Even though most surrogate markers for CVD risk showed a significant difference between one of the KD-groups and controls in most studies, the pathophysiological mechanism behind these changes following KD is still unclear. In fact, post-mortem studies have failed to show atherosclerotic changes, even in affected coronary arteries, [57]. In this post-mortem study, active remodeling of the coronary arterial wall could be identified years after the acute stage of the disease, potentially indicating that a distinct cardiovascular process, other than atherosclerosis, may be held responsible for an increased CVD risk following the early period of acute vascular inflammation in KD. Prolonged (low-grade) inflammation as suggested by the presence of increased levels of inflammatory markers such as (high-sensitivity) CRP are believed to be associated with the occurrence of cardiovascular events in adults [58,59]. However, controversy exists as to whether patient with KD have a continued low-grade inflammation years after the disease [12,21,24,32].

A pathophysiological mechanism responsible for the changes in the vasculature in KD, both in the coronary and the peripheral arteries, has yet to be elucidated. Whether persistent low-grade inflammation or genetic factors may play a role in this “KD-vasculopathy” and in remodelling of the arterial wall is as yet unclear.

### Limitations

Some limitations of our study have to be mentioned. First, we found substantial heterogeneity between studies. Because of this heterogeneity, we could not pool most of the results from the original studies. Hence, conclusions can only be drawn from a summary of these studies without a statistical finding.

Although we tried to find the source of heterogeneity by performing meta-regression analyses, we could not find factors for all surrogate markers. For both cIMT and SI, we could include less than 10 studies in the meta-regression analyses; it is questionable whether such numbers are large enough because of a lack of power.

For this review we considered patients who ever had CAA as CAA-positive. However, CAA range from small to giant, reflecting the severity of the original vasculitis and it may thus not be appropriate to combine all CAA-positive patients into one group.

None of the included studies reported on long-term longitudinal data as they were all cross-sectional or very short-term cohort studies. Long-term follow-up is necessary to investigate the course of the surrogate markers over time as well as the natural course of the disease and to predict CVD risk at a later age.

### Conclusion

This systematic review and meta-analyses suggests that surrogate markers for CVD risk in patients after KD are increased in CAA-positive but not in CAA-negative patients. The results have to be interpreted with care due to methodological limitations and high heterogeneity between studies which prevents the possibility of data pooling. However, these findings might indicate that CAA-positive patients should be monitored and counselled for CVD in later life. Long-term follow-up of former KD patients is needed to confirm our results.

## Supporting Information

S1 PRISMA Checklist PRISMA checklist for systematic reviews and meta-analyses. (DOC)

S1 File Electronic MEDLINE search strategy. (DOC)

S1 Table Quality assessment criteria. (DOCX)

S2 Table Quality assessment per study. (DOCX)
