# Supplementary material for: Peripheral Endothelial (Dys)Function, Arterial Stiffness and Carotid Intima-Media Thickness in Patients after Kawasaki Disease: A Systematic Review and Meta-Analyses
Source: PLoS One. 2015 Jul 10;10(7):e0130913. doi: 10.1371/journal.pone.0130913 (PMC4498761; doi:10.1371/journal.pone.0130913)
Supplement: S1 File — (DOC) [file pone.0130913.s002.doc]

**Medline electronic search strategy**

("Carotid Intima-Media Thickness"[Mesh] OR intima media thick* [tiab] OR IMT [tiab] OR intimal thick* [tiab] OR myointimal thick* [tiab] OR "Vascular Stiffness"[Mesh] OR stiffness OR stiffening OR endothelial dysfunction [tiab] OR endothelial function [tiab] OR flow mediated dilat* [tiab] OR flow-mediated dilat* [tiab] OR FMD [tiab] OR artery dilat* [tiab] OR artery reactivity dilat* [tiab] OR "Compliance"[Mesh] OR Distensibility [tiab] OR "Pulse Wave Analysis"[Mesh] OR pulse wave analys* [tiab] OR pulse wave velocity [tiab] OR pulse-wave velocity [tiab] OR PWV [tiab] OR "Atherosclerosis"[Mesh] OR atherosclerosis [tiab] OR atherosclerotic [tiab] OR vascular ultrasonography [tiab] OR vascular elasticity [tiab] OR peripheral arterial tonometry [tiab] OR endo pat [tiab] OR cardiovascular risk [tiab]) AND ("Mucocutaneous Lymph Node Syndrome"[Mesh] OR kawasaki [tiab])
