# Supplementary material for: Peripheral Endothelial (Dys)Function, Arterial Stiffness and Carotid Intima-Media Thickness in Patients after Kawasaki Disease: A Systematic Review and Meta-Analyses
Source: PLoS One. 2015 Jul 10;10(7):e0130913. doi: 10.1371/journal.pone.0130913 (PMC4498761; doi:10.1371/journal.pone.0130913)
Supplement: S1 Table — (DOCX) [file pone.0130913.s003.docx]

**Supplemental table S1: Quality assessement criteria (adjusted Newcastle-Ottawa criteria)**

|  | Selection | | | | Comparability | | Outcome | |
| --- | --- | --- | --- | --- | --- | --- | --- | --- |
|  | **Patient group** | **Representa-tiveness** | **Selection of controls** | **Definition of controls** | **Identification** | **Adjustment** | **Description** | **Blinding** |
| Adequate (++) | *If* description of amount of children with CAA  *And* time of measurement (worst-ever or current/ when echocardiographies were performed)  *And* criteria for CAA mentioned | *If* all eligible cases with KD over a defined period of time  *Or* all cases with KD in a defined hospital or clinic, group of hospitals, health organisation, or an appropriate sample of those cases (e.g. random sample). | Community-based controls, hospital-based controls; clearly stated from where.  *Or* siblings of KD patients | *No* evidence of disease history in participant  *And* no family history of premature ischemic disease, or hypertension, hypercholesterolemia or other factors that might affect vascular function | Identification of factors/possible confounders known to influence the surrogate markers including age, gender, blood pressure and BMI. | Adjustment for factors in outcome. | Clear description:  cIMT: protocol, site, machine  FMD: protocol, site, machine  PWV: site, method, machine  PA: site, method and machine  SI: site, method of calculation and machine | Outcome measured in a blinded manner. |
| Moderate  (+) | *If* description of amount of children with CAA  *And* time of measurement (worst-ever or current) |  | Controls from a different group (e.g. family and friend of hospital staff).  *Or* controls from a clearly described group, but unclear whether from the same community/hospital. | No evidence of disease history in participant. | Identification of age and gender. | If multiple regression analyses was performed in both groups, no factors were of influence on IMT, FMD (or NMD), PWV or PAT. | Incomplete description: description of protocol, but not of used machine. |  |
| Incomplete  (-) | No description | No description | No description | No mention of (family) history of vascular or endothelial abnormalities. | No identification of age and gender. | No correction  *Or* factors shown to be of influence on surrogate marker or multiple regression analyses only performed in either patients or control group | No clear description | Outcome not measured in a blinded manner |
