# Supplementary material for: Peripheral Endothelial (Dys)Function, Arterial Stiffness and Carotid Intima-Media Thickness in Patients after Kawasaki Disease: A Systematic Review and Meta-Analyses
Source: PLoS One. 2015 Jul 10;10(7):e0130913. doi: 10.1371/journal.pone.0130913 (PMC4498761; doi:10.1371/journal.pone.0130913)
Supplement: S2 Table — (DOCX) [file pone.0130913.s004.docx]

**Supplemental table S2: Quality assessment per study.**

|  | Selection | | | | Comparability | | Outcome | | |  | Total |
| --- | --- | --- | --- | --- | --- | --- | --- | --- | --- | --- | --- |
| Author | Patient group | Representativeness | Selection of controls | Definition of controls | Identification | Correction | Description | Blinding |  | |  |
| Dhillon, 1996 | + | - | + | ++ | + | - | ++ | ++ | 4/1/4 | | 9 |
| Silva, 2001 | ++ | ++ | - | - | ++ | - | + | - | 4/2/1 | | 7 |
| Noto, 2001 | + | - | - | ++ | ++ | - | ++ | ++ | 3/2/4 | | 9 |
| Deng, 2002 | ++ | - | - | + | + | - | ++ | ++ | 3/1/4 | | 8 |
| Cheung, 2004 Heart | + | - | - | - | ++ | - | ++ | - | 1/2/2 | | 5 |
| Cheung, 2004 Ped r | + | - | ++ | - | ++ | - | + | - | 3/2/1 | | 6 |
| Cheung, 2004 JACC | + | - | ++ | - | ++ | - | + | - | 3/2/1 | | 6 |
| Ikemoto, 2005 | ++ | - | + | + | ++ | - | ++ | ++ | 4/2/4 | | 10 |
| Kadono, 2005 | ++ | - | ++ | + | + | - | ++ | ++ | 5/1/4 | | 10 |
| Borzutzky, 2007 | - | - | - | + | ++ | - | + | - | 1/2/1 | | 4 |
| McCrindle, 2007 | + | ++ | ++ | ++ | ++ | - | ++ | - | 7/2/2 | | 11 |
| Cheung, 2007 | + | - | ++ | - | ++ | + | ++ | ++ | 3/2/4 | | 9 |
| Huang, 2008 | + | - | ++ | + | ++ | - | ++ | ++ | 4/2/4 | | 10 |
| Niboshi, 2008 | + | - | + | + | ++ | - | ++ | - | 3/2/2 | | 7 |
| Cheung, 2008 | ++ | - | ++ | - | ++ | +/- | ++ | - | 4/3/2 | | 9 |
| Liu, 2009 | ++ | - | ++ | + | ++ | - | ++ | - | 5/2/2 | | 9 |
| Gupta, 2009 | + | - | - | ++ | ++ | - | + | ++ | 3/2/3 | | 8 |
| Lee, 2009 | ++ | - | - | - | - | - | ++ | ++ | 2/0/4 | | 6 |
| Noto, 2009 | + | ++ | + | - | ++ | - | ++ | ++ | 4/2/4 | | 10 |
| Duan, 2011 | ++ | - | + | - | + | - | ++ | - | 3/1/2 | | 6 |
| Noto, 2012 | + | - | - | - | ++ | - | ++ | - | 1/2/2 | | 5 |
| Pinto, 2013 | + | - | + | ++ | + | - | ++ | - | 4/1/2 | | 7 |
| Tobayama, 2013 | ++ | - | - | ++ | ++ | - | ++ | - | 4/2/2 | | 8 |
| Ishikawa, 2013 | ++ | - | ++ | + | ++ | - | ++ | ++ | 5/2/4 | | 11 |
| Laurito, 2013 | + | - | - | ++ | ++ | - | ++ | - | 3/2/2 | | 7 |
| Oguri, 2013 | ++ | ++ | + | - | ++ | - | ++ | ++ | 5/2/4 | | 11 |
| Selamet Tierney, 2013 | ++ | - | ++ | + | ++ | - | ++ | - | 5/2/2 | | 9 |
| Singh-Meena, 2013 | + | ++ | ++ | - | + | - | ++ | ++ | 5/1/4 | | 10 |
| Duan, 2014 | ++ | - | ++ | - | ++ | - | ++ | ++ | 4/2/4 | | 10 |
| Cho, 2014 | + | - | ++ | + | ++ | - | + | - | 4/2/1 | | 7 |
